# SARS-CoV-2 infection of monocytes: balancing acts of antibodies and inflammasomes

**DOI:** 10.1038/s41392-022-01112-w

**Published:** 2022-07-23

**Authors:** R. Camille Brewer, William H. Robinson, Tobias V. Lanz

**Affiliations:** 1 Division of Immunology and Rheumatology, Department of Medicine, Stanford University School of Medicine, 269 Campus Drive, Stanford, CA 94305 USA; 2 Geriatric Research, Education, and Clinical Centers (GRECC), VA Palo Alto Health Care System, 3801 Miranda Ave, Palo Alto, CA 94304 USA

**Keywords:** Infection, Inflammation, Innate immunity

**Fig. 1:**
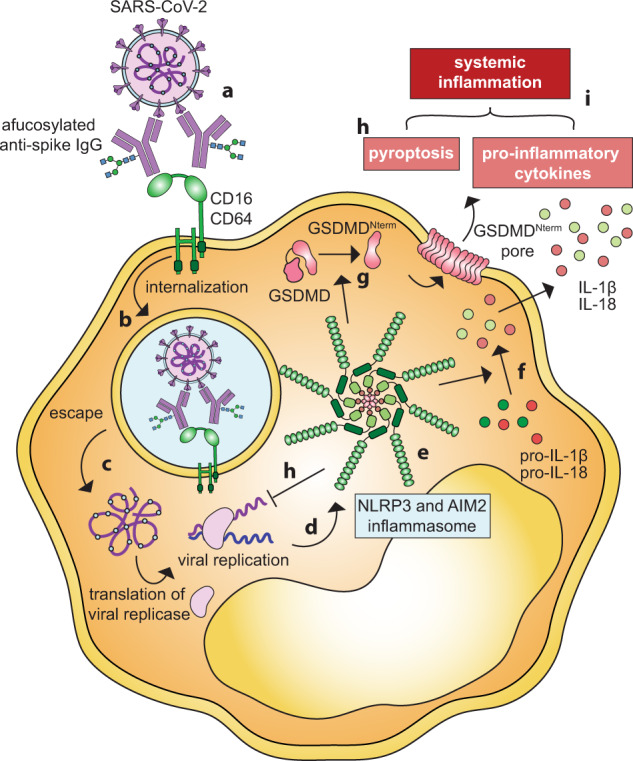
Antibody-mediated SARS-CoV-2 infection of monocytes via FcγRs. **a** Immune complexes composed of afucosylated anti-SARS-CoV-2 IgG and virus infect monocytes via FcγRIIIa and FcγRI, which initiates **b** phagocytosis. **c** SARS-CoV-2 escapes degradation via the phagolysosome and initiates replication in the cytoplasm. **d** Replication of SARS-CoV-2 activates NLRP3 and AIM2 leading to the formation of **e** the NLRP3 and AIM2 inflammasome complex. **f** Inflammasome activation induces cleavage of pro-IL-1β and pro-IL-18 resulting in the release of pro-inflammatory cytokines IL-1β and IL-18. **g** Gasdermin D (GSDMD) is cleaved and inserted into the membrane instigating pyroptosis. **h** Inflammasome activation and pyroptotic cell death halt SARS-CoV-2 replication. **i** Secretion of IL-1β and IL-18 and pyroptosis contribute to systematic inflammation, associated with severe COVID-19. GSDMD amino-terminal cell death domain (GSDMD^Nterm^)

In a recent study in *Nature*, Junqueira et al. provided evidence that severe acute respiratory syndrome coronavirus 2 (SARS-CoV-2) infects human monocytes and lung macrophages.^1^ The infection is antibody-mediated via Fc-receptors (FcγRs), and activates NLRP3 and AIM2 inflammasomes, which in turn induce pyroptosis and halt viral replication. Infected monocytes secrete the pro-inflammatory cytokines interleukin-1β (IL-1β) and IL-18, which contribute to systemic inflammation and severe coronavirus disease 19 (COVID-19) (Fig. 1).

Since its initial description in December 2019, our understanding of COVID-19 has expanded from a respiratory illness to a multi-system disease. We have since understood that SARS-CoV-2 can directly infect a variety of cells and tissues beyond the lung, and that concomitant systemic immune responses contribute to disease severity. Several important questions remain, including how the virus enters cell types devoid of the viral entry receptor ACE2, if it survives and replicates in each cell type, and how the infected cells contribute to COVID-19 symptoms and severity.

Junqueira et al. demonstrated that approximately 10% of blood monocytes and 8% of lung macrophages in COVID-19 patients are infected with SARS-CoV-2.^1^ Monocytes do not express ACE2, the canonical viral entry receptor for SARS-CoV-2. Instead, viral entry is facilitated by FcγRIIIa (CD16a) and FcγRI (CD64) and requires opsonization of the virus by host antibodies. This might suggest that anti-SARS-CoV-2 antibodies could elicit antibody-dependent enhancement (ADE), a dreaded immunological phenomenon where anti-viral antibodies boost disease severity, either by promoting viral cell entry via FcγRs or by inducing excessive Fc-mediated effector functions.^2^ ADE is best documented in Dengue virus infection. In Dengue, non-neutralizing antibodies aid viral uptake into macrophages, leading to productive infections, increased viral loads, and aggravated disease. Fortunately, SARS-CoV-2 differs from Dengue in several ways: (i) FcγRIIa mediates ADE in Dengue, whereas FcγRIIIa and FcγRI facilitate monocyte infection with SARS-CoV-2. (ii) ADE-mediated infection with Dengue results in productive viral replication in macrophages, whereas SARS-CoV-2 replication in human monocytes is aborted by inflammasome activation and pyroptosis. (iii) Non-neutralizing ADE-facilitating antibodies in Dengue can originate from prior infections with differing virus strains as well as from experimental vaccines. In contrast, sera from individuals vaccinated against SARS-CoV-2 did not promote monocyte infection,^1^ possibly due to the vaccines’ narrow focus including only the spike antigen. This observation is in line with SARS-CoV-2 vaccine studies, which demonstrated the effectiveness of the vaccines with no evidence for ADE. The authors note, however, that the mechanism could play a role in convalescent plasma therapies, which were largely unsuccessful in the treatment of COVID-19.

Interestingly, IgG afucosylation influences the efficacy of monocyte infection. Afucosylation is a modification of the IgG-Fc domain that increases its affinity for FcγRIIIa.^3^ Plasma from COVID-19 patients with high levels of afucosylated anti-spike IgG facilitates monocyte infection more strongly than plasma with low levels of afucosylated IgG. In line with this observation, severe COVID-19 patients exhibit elevated levels of afucosylated anti-SARS-CoV-2 IgG, whereas IgG from vaccinated individuals are mostly fucosylated, and plasma from vaccinated individuals does not promote infection of monocytes.^1,3^

SARS-CoV-2 infection of monocytes and macrophages activates NLRP3 and AIM2 inflammasomes.^1,4^ Inflammasomes are multiprotein complexes of receptors and adapter molecules which, in response to pathogens, activate caspases to initiate the processing and secretion of inflammatory cytokines and induce pyroptosis. The inflammasome response is necessary to halt SARS-CoV-2 replication in monocytes and macrophages. The secreted inflammatory cytokines, in particular interleukin-1β (IL-1β) and IL-18, act as warning signals to surrounding cells by inducing interferons and activating the NF-κB pathway, thereby further limiting the spread of the virus. However, IL-1β and IL-18, together with the caspase-1 substrate gasdermin D (GSDMD) and lactate dehydrogenase (LDH, released during pyroptosis), have been associated with severe courses of COVID-19.^1,5^ The current study identifies infected monocytes as the likely source of these inflammatory drivers of disease and suggests that enhanced inflammasome activation contributes to excessive inflammation and severe COVID-19. The causes of excessive inflammasome responses in certain patients remain unclear. However, conditions that have been identified as risk factors for severe COVID-19, including type 2 diabetes and obesity, are often associated with dysregulated NLRP3 inflammasome responses.

The same authors showed in a humanized mouse model that pharmacological inhibition of inflammasomes with caspase-1 and NLRP3 inhibitors decreased levels of pro-inflammatory cytokines and reversed lung tissue damage caused by SARS-CoV-2 infection.^4^ However, viral loads are elevated in the lungs of mice treated with inflammasome inhibitors and in-vitro experiments indicate that inflammasome inhibition allows SARS-CoV-2 to successfully propagate in lung macrophages.^4^ Therapies aiming at inflammasome inhibition might therefore be a double-edged sword, as such might prevent cytokine-mediated tissue damage but also increase viral loads in the lungs and could enable circulating monocytes to disseminate the virus. Nevertheless, the approach could be promising in select patients with severe disease and strong inflammasome activation, indicated by elevated levels of IL-1β, IL-18, GSDMD, and LDH. Clinical trials using NLRP3 inflammasome inhibitors disulfiram and dimethyl fumarate are ongoing.

In summary, Junqueira et al. demonstrate antibody-mediated infection of monocytes and macrophages by SARS-CoV-2, which induces inflammasome activation. While productive replication of the virus is halted by pyroptosis, the inflammatory cytokine response likely contributes to severe COVID-19. Targeting the inflammasome is a promising strategy for novel therapies for severe COVID-19, but caution is warranted due to its opposing protective and aggravating functions in SARS-CoV-2 pathology.
